# The functions of FXYD family members in human health and disease

**DOI:** 10.1016/j.gendis.2025.101847

**Published:** 2025-09-10

**Authors:** Xi Li, Min Long, Shangwei Zhong, Jun-Li Luo

**Affiliations:** a The Cancer Research Institute and the Second Affiliated Hospital, Hengyang Medical School, University of South China, Hengyang, Hunan 421001, China; b MOE Key Lab of Rare Pediatric Diseases, Hengyang Medical School, University of South China, Hengyang, Hunan 421001, China; c Hunan Provincial Key Laboratory of Basic and Clinical Pharmacological Research of Gastrointestinal Cancer, University of South China, Changsha, Hunan 421001, China; d National Health Commission Key Laboratory of Birth Defect Research and Prevention, Hunan Provincial Maternal and Child Health Care Hospital, University of South China, Changsha, Hunan 410008, China

**Keywords:** FXYD family, Human health and disease, Ion channel, Na^+^/K^+^-ATPase, NKA

## Abstract

The FXYD family (FXYD domain-containing ion transport regulators) proteins consist of short, single-pass transmembrane proteins that primarily regulate the Na^+^/K^+^-ATPase (NKA) pump, a key player in maintaining cellular ion homeostasis. Ranging from 60 to 160 amino acids in length, FXYD proteins display tissue-specific expression patterns and influence not only NKA activity but also the function of other ion channels, including potassium, sodium, and chloride channels. These proteins interact with NKA in diverse ways, modulating its activity to meet the specific needs of different tissues. In addition to their physiological roles, FXYD proteins are implicated in the development and progression of various diseases, such as cancer, cardiovascular disorders, renal diseases, and neurological conditions. This review offers an overview of the structures, biological functions, and molecular mechanisms through which FXYD proteins regulate ion transport. Furthermore, we explore their emerging roles in disease pathogenesis and discuss potential therapeutic strategies for targeting FXYD proteins in disease management.

## Introduction

**Figure 1:**
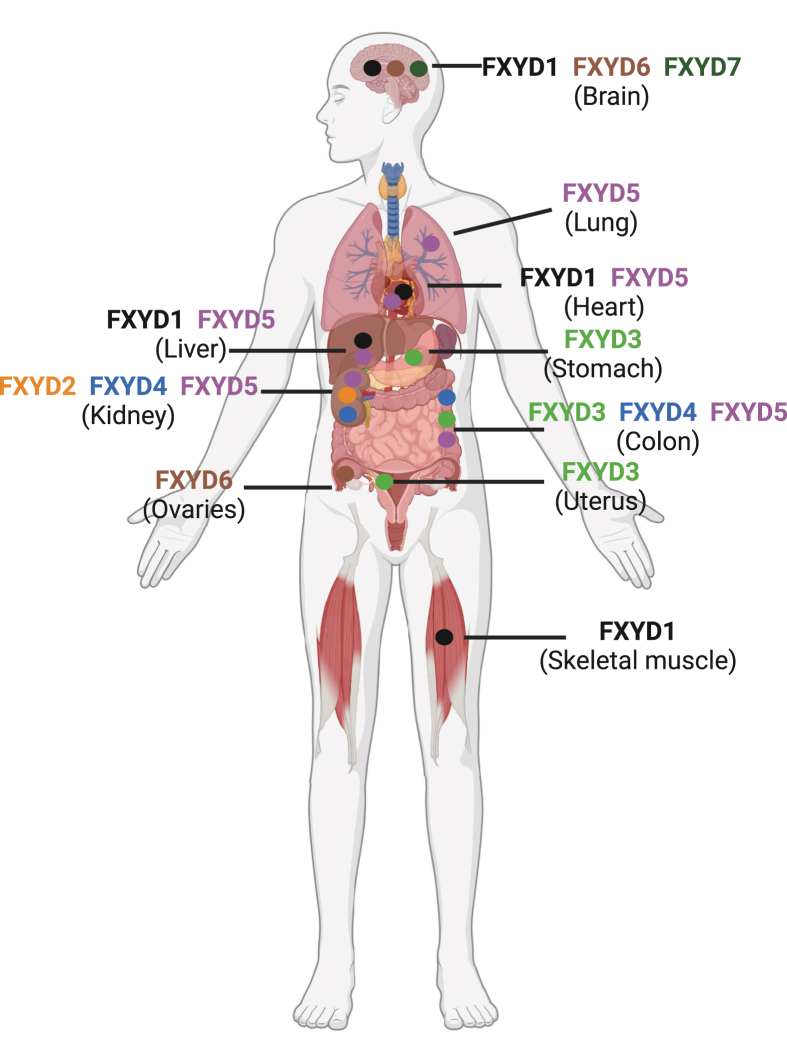
The diagram of the distribution of FXYD family members across tissues. FXYD1 is found in the brain, heart, liver, and muscle. FXYD5 is expressed in the lung, heart, liver, kidney, and colon. FXYD2/4 localize mainly in the kidney and colon, while FXYD3 appears in the colon, stomach, and uterus. FXYD6 is enriched in the brain and ovaries, and FXYD7 is primarily in the brain.

The FXYD family (FXYD domain-containing ion transport regulators) proteins consist of seven distinct proteins, each characterized by a conserved 35-amino-acid motif known as the FXYD domain, which is central to their functions. These proteins play a crucial role in regulating the activity of Na^+^/K^+^-ATPase (NKA), a vital membrane pump responsible for maintaining cellular ion gradients and homeostasis. Each FXYD protein exhibits tissue-specific expression, contributing to diverse roles in various tissues, including the heart, kidney, brain, and gastrointestinal system.^1^, ^2^, ^3^, ^4^, ^5^ For example, FXYD1 is found in muscle and various other types of tissues,^6^, ^7^, ^8^ FXYD2 is primarily in the kidney,^3^ FXYD3 is highly expressed in the stomach, colon, and uterus,^9^^,^^10^ FXYD4 is also in the kidney, FXYD5 is in various epithelial tissues,^11^ FXYD6 is in auditory tissues,^12^ and FXYD7 is in neurons and glial cells^13^ (Fig. 1).

Emerging evidence highlights the involvement of FXYD proteins in various physiological processes, including ion transport, neuronal activity, cell adhesion, and migration.^14^, ^15^, ^16^, ^17^ Notably, altered expression levels of these proteins have been associated with various diseases, particularly cancer, where they may either promote or suppress tumorigenesis depending on the specific context.^18^, ^19^, ^20^, ^21^ For instance, elevated levels of FXYD3 and FXYD5 have been linked to aggressive tumor behavior in renal cancer, breast cancer, and other cancers, while FXYD2 has demonstrated tumor-suppressive properties in certain malignancies.^18^, ^19^, ^20^, ^21^

In this review, we provide an overview of the structures, biological functions, and molecular mechanisms by which FXYD proteins regulate ion transport. We also explore the role of the FXYD family proteins in the pathogenesis of various diseases, including cancer, neurological disorders, kidney diseases, cardiovascular conditions, and gastrointestinal disorders. This review synthesizes the current understanding of FXYD proteins in both health and disease, emphasizing their functional mechanisms and potential as therapeutic targets.

## Structural characteristics of FXYD proteins

### The common features of FXYD proteins

**Figure 2:**
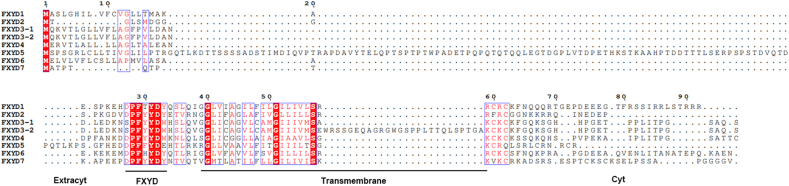
The sequence alignment of human FXYD proteins, highlighting conserved domains and structural features. Key regions are annotated: Extracyt, FXYD, Transmembrane, and Cyt. Sequence numbering is provided above the alignment. Variations in sequence length and domain organization reflect functional diversity among FXYD isoforms.

FXYD proteins are defined by a conserved 35-amino-acid domain known as the FXYD motif, which is crucial for their functions and regulatory interaction with NKA.^22^ The motif is composed of proline (P), phenylalanine (F), tyrosine (Y), and aspartic acid (D), and may also include additional conserved residues that enhance its regulatory function.^22^ This motif facilitates interactions with the alpha and beta subunits of NKA, enabling FXYD proteins to influence the pump's affinity for ATP and its ion transport kinetics.^23^ Each protein of the FXYD family has distinct residues flanking the conserved motif, which contribute to their specific regulatory effects and tissue-specific expression patterns^24^ (Fig. 2).

FXYD proteins contain a single transmembrane (TM) domain that anchors them to the cell membrane, enabling close interaction with membrane-associated ion transporters.^23^ This hydrophobic TM region allows FXYD proteins to integrate into the lipid bilayer, facilitating stable interactions with NKA. The orientation of the TM domain allows FXYD proteins to modulate pump activity from within the membrane, thus contributing to the maintenance of cellular ion gradients.^24^

FXYD proteins bind directly to NKA, typically interacting with the alpha subunit, and occasionally with the beta subunit, of the pump.^24^ The conserved FXYD motif is essential for binding affinity and specificity, enabling different FXYD proteins to uniquely modulate NKA function in response to varying cellular conditions. FXYD proteins alter the NKA pump's kinetics by modifying its conformation, adjusting ion affinity, and fine-tuning the pump's activity to meet the tissue-specific needs of organs such as the heart, kidney, and nervous system.^17^^,^^24^, ^25^, ^26^ These structural features allow FXYD proteins to regulate the NKA's activity, supporting essential processes like maintaining membrane potential, regulating cell volume, and adapting to changes in osmolarity.^17^^,^^24^, ^25^, ^26^

### Structural differences among FXYD family proteins

FXYD1 is a 72-amino-acid protein with the characteristic PFXYD motif at its extracellular N-terminus, a single TM domain, and a cytoplasmic C-terminal tail containing three phosphorylation sites.^27^^,^^28^ It is the only protein of the FXYD family known to have multiple phosphorylation sites, which can be individually or collectively modified by kinases and dephosphorylated by phosphatases.^29^^,^^30^ FXYD2 is encoded by a gene spanning over 9 kb and comprising seven exons. The first translated exon undergoes alternative splicing to produce two variants, FXYD2a and FXYD2b, which differ in their N-terminal sequences.^31^^,^^32^ FXYD3, also known as Mat-8, serves as a tissue-specific modulator of NKA. The FXYD3 gene produces two primary transcripts, FXYD3a and FXYD3b, with the latter variant differing by a 26-amino-acid insertion after the TM domain. FXYD4, also called channel-inducing factor (CHIF), is a small (8 kDa) single-span membrane protein. It features a conserved 35-amino-acid sequence beginning with PFXYD, containing seven invariant and six highly conserved amino acids.^33^ FXYD5, also known as dysadherin or RIC, is a 178-amino-acid single-span TM protein with a conserved 35-amino-acid sequence involved in ion transport through its interaction with NKA. Structurally and functionally distinct from other family proteins, FXYD5 has an unusually long extracellular domain of over 140 amino acids, including a cleavable signal peptide.^11^ FXYD6 (phosphohippolin) is a 10 kDa membrane protein, a recent addition to the FXYD family, and is known to regulate NKA by interacting with the β1 subunit.^34^ FXYD7 is a 14 kDa, brain-specific FXYD protein that lacks a cleavable signal sequence^13^ (Fig. 2).

## Physiological roles of FXYD proteins

### Ion transport and adaptation to cellular stress and homeostatic changes

The FXYD protein family plays a critical role in ion transport and cellular homeostasis by modulating the activity of the NKA pump, which is essential for maintaining cellular ion gradients. NKA moves sodium (Na^+^) and potassium (K^+^) ions across the plasma membrane by exporting three Na^+^ ions from the cell and importing two K^+^ ions per cycle. This process establishes the electrochemical gradients required for vital physiological functions, such as maintaining membrane potential, regulating cell volume, and supporting the secondary active transport of other molecules.^17^^,^^35^ FXYD proteins regulate NKA by modifying its kinetic properties, including its affinity for Na^+^ and K^+^ ions, as well as its sensitivity to ATP. By binding to NKA, FXYD proteins alter the pump's conformation, enabling tissue-specific adaptations in pump activity that meet the unique needs of various organs. This modulation allows cells to maintain precise control over intracellular ion concentrations and osmotic balance^15^^,^^24^^,^^25^ (Table 1).

**Table 1:** Summary of FXYD protein characteristics.

Protein Name	Also known as	Signal Peptide	Expression in tissues	Association with NKA	lon affinities
FXYD1	PLM	Yes	Heart, skeletal muscle	β1	Na↓ K↓
FXYD2	γ subunit	NO	Kidney	α1	Na↓ K-
FXYD3	MAT8 PLML	Yes	Stomach, colon	α1,β1	Na ↓K↓
FXYD4	CHIF	Yes	Kidney	α1,β1	Na↑ K-
FXYD5	RIC, dyshaderin	Yes	Brain, liver	β1	Na↑ K↓
FXYD6	–	Yes	Brain	β1	Na– K↓
FXYD7	–	NO	Brain	α1,β	Na– K↓

Furthermore, FXYD proteins enable cells to adjust to stressors, such as osmotic shifts, hypoxia, and metabolic changes,^22^^,^^36^^,^^37^ by modulating NKA activity in response to these conditions. In cells exposed to high osmolarity, for example, FXYD proteins help retain potassium to mitigate the effects of dehydration and preserve cellular volume. In response to metabolic changes or hormonal signals, such as aldosterone in the kidney,^24^^,^^38^^,^^39^ FXYD proteins can either increase or decrease NKA activity to align with the body's regulatory needs, such as enhancing salt retention during low-salt conditions.^24^ By modulating NKA activity, FXYD proteins are essential for maintaining ion gradients and cell volume, thereby supporting various physiological functions and ensuring homeostasis across tissues. Their tissue-specific expression and precise regulation make FXYD proteins pivotal in organ-specific functions and overall health.

### Tissue-specific regulation

**Figure 3:**
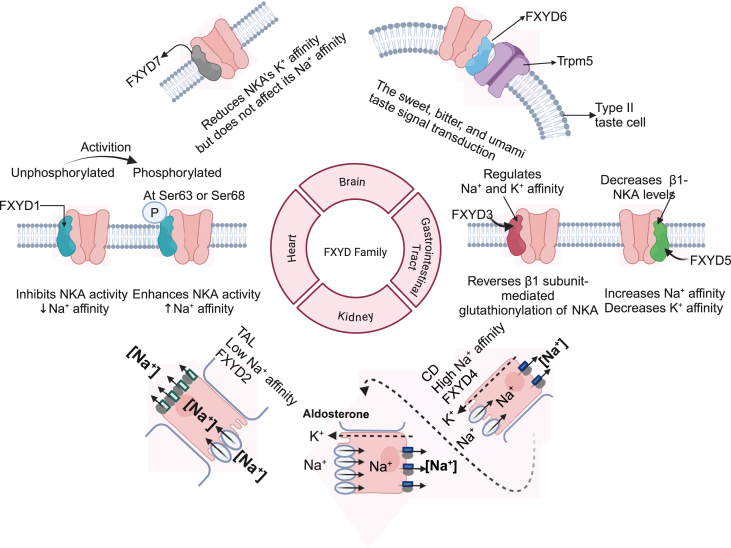
The diverse regulatory roles of FXYD family proteins in modulating NKA activity across different tissues. FXYD1, highly expressed in the heart, regulates both Na^+^ and K^+^ affinity of NKA. Its phosphorylation at Ser63 or Ser68 enhances NKA activity, whereas the unphosphorylated form inhibits NKA. FXYD2, primarily localized in the thick ascending limb of Henle's loop (TAL), increases Na^+^ affinity and lowers K^+^ affinity, contributing to sodium reabsorption; it is regulated by aldosterone and local sodium concentrations. FXYD3 modulates the expression of NKA β1 subunits and reverses glutathionylation, impacting redox-sensitive regulation of pump activity. FXYD4 exhibits high Na^+^ affinity and plays a role in renal sodium handling, particularly under low sodium or high aldosterone conditions. FXYD5 is associated with increased Na^+^ affinity and reduced K^+^ affinity. FXYD6 is co-expressed with the taste receptor channel TRPM5 and contributes to taste signal transduction for sweet, bitter, and umami stimuli. FXYD7, predominantly expressed in neurons, reduces NKA's affinity for K^+^ but does not affect Na^+^ binding.

Each protein of the FXYD family exhibits a distinct expression pattern across different tissues, allowing for specialized regulation of ion transport that is tailored to the needs of specific organs (Fig. 3). In the heart, FXYD1 regulates NKA to maintain ionic balance, which is crucial for muscle contractility and electrical stability, both of which are necessary for normal heart rhythm.^15^^,^^36^ When unphosphorylated, FXYD1 inhibits NKA activity by reducing its affinity for intracellular Na^+^, whereas phosphorylation at Ser63 or Ser68 reverses this effect, activating NKA.^40^, ^41^, ^42^ Peroxiredoxin 6 (Prdx6) regulates the depalmitoylation of phospholemman (PLM) in a glutathione-dependent manner, modulating sodium pump activity by removing palmitic acid from PLM via a thioesterase mechanism, which has implications for cellular glutathione status^43^ (Fig. 3). FXYD1 also interacts with the NKA α1 subunit, contributing to brain development, maintaining neuroepithelial integrity, regulating neuroepithelial permeability, and supporting cerebrospinal fluid production.^44^ Additionally, FXYD1 may play a role in the neuroendocrine regulation of female puberty by modulating the excitability of gonadotropin-releasing hormone (GnRH) neurons.^45^

In the kidney, FXYD2 and FXYD4^15^^,^^38^^,^^46^ regulate NKA in renal epithelial cells, which is essential for sodium reabsorption and potassium excretion, processes that are critical for blood pressure regulation and electrolyte balance. FXYD2, known as the γ subunit of NKA,^32^ associates with the NKA α and β subunits in the kidney. It is worked in the proximal tubule and in the medullary part of the thick ascending limb of Henle's loop. As an auxiliary subunit, FXYD2 not only modulates NKA's cation transport properties but also influences the voltage dependence of K^+^ activation and alters NKA's apparent affinity for Na^+^ and K^+^ .^3^ FXYD4, which is selectively expressed in the collecting ducts (CD), specifically binds to the NKA α subunit, enhancing the enzyme's rate constant and selectively increasing its affinity for cytoplasmic Na^+^, without affecting its affinity for extracellular K^+^ or the enzyme's maximal rate.^2^^,^^47^ FXYD4 promotes a two-to-three-fold increase in Na^+^ affinity, whereas FXYD2 decreases Na^+^ affinity. These opposing effects help define NKA activity across different nephron segments, thus supporting tubular Na^+^ reabsorption^48^^,^^49^ (Fig. 3).

In the brain, FXYD6 and FXYD7^12^^,^^50^^,^^51^ modulate neuronal excitability by adjusting ion gradients, which is essential for proper synaptic signaling and protection against excitotoxicity. FXYD6 is expressed in type II taste cells, where it coexists with transient receptor potential cation channel subfamily M member 5 (Trpm5) and NKA β1, suggesting a role in taste signal transduction,^34^ and in the endolymphatic sac, where it may help regulate endolymph volume.^52^ Researchers have also investigated the role of conserved and FXYD7-specific amino acids in the cellular routing of FXYD7 and its association with and regulation of NKA.^26^^,^^53^^,^^54^ They found that conserved Gly40 and Gly29 of FXYD7, located on the same face of the TM helix, are involved in both the association with and regulation of NKA.^54^ FXYD7 reduces NKA's K^+^ affinity but does not affect its Na^+^ affinity^13^ (Fig. 3).

In tissues such as the gastrointestinal tract and tumors, FXYD3 and FXYD5^4^^,^^55^, ^56^, ^57^ may influence pH and cell adhesion properties, which in turn affect ion transport (Table 1). FXYD3b is primarily expressed in undifferentiated cells, while FXYD3a is more abundant in differentiated cells. Both isoforms can be co-immunoprecipitated with NKA antibodies, suggesting a direct interaction with NKA.^58^ FXYD3 has been identified as a downstream target of transforming growth factor-beta (TGF-β) signaling via zinc finger E-box binding homeobox 1 (ZEB1, also termed δEF1), though it is not directly involved in epithelial-to-mesenchymal transition.^59^ FXYD3 regulates NKA by modulating its affinity for Na^+^ and K^+^, and can reverse β1 subunit glutathionylation of NKA, thereby mitigating oxidative inhibition of the pump.^39^^,^^60^ FXYD5, on the other hand, decreases β1-NKA levels, doubling the enzyme's Na^+^ affinity and reducing its K^+^ affinity by 60%, which may help moderate elevated intracellular Na^+^ or low K^+^ levels without altering NKA cell numbers.^5^

## FXYD proteins in human diseases

### FXYD proteins in cancer

FXYD proteins are recognized as key modulators of tumorigenesis, influencing cell proliferation, migration, invasion, and the formation of the tumor microenvironment. The FXYD family members exhibit distinct roles in tumor progression. FXYD5^20^^,^^61^ and FXYD6^62^, ^63^, ^64^ demonstrate pro-oncogenic effects, whereas FXYD2^21^^,^^65^ primarily functions as a tumor suppressor. Notably, FXYD3^66^^,^^67^ displays a dual regulatory role, manifesting both tumor-promoting and tumor-suppressive effects depending on cellular context.

### Tumor-promoting effects

FXYD5 is highly expressed in epithelial tissues such as the lung, kidney, and intestine, where it regulates chemokine production, cell adhesion, and junction integrity. By inhibiting E-cadherin, it reduces cell–cell adhesion and increases metastatic potential in liver cancer, pancreatic cancer, and breast cancer.^68^ Clinical studies associate FXYD5 overexpression with poor prognosis and immune evasion, suggesting its value as a prognostic biomarker and therapeutic target.^61^ FXYD5 promotes sorafenib resistance in hepatocellular carcinoma (HCC) by activating the protein kinase B (Akt)/mammalian target of rapamycin (mTOR) signaling pathway, making it a potential therapeutic target for overcoming chemoresistance.^69^ Its aberrant O-glycosylation produces cancer-specific epitopes, making it an attractive target for immunotherapy, evidenced by the monoclonal antibody 6C5, which specifically recognizes a Tn-glycopeptide in FXYD5.^70^

**Figure 4:**
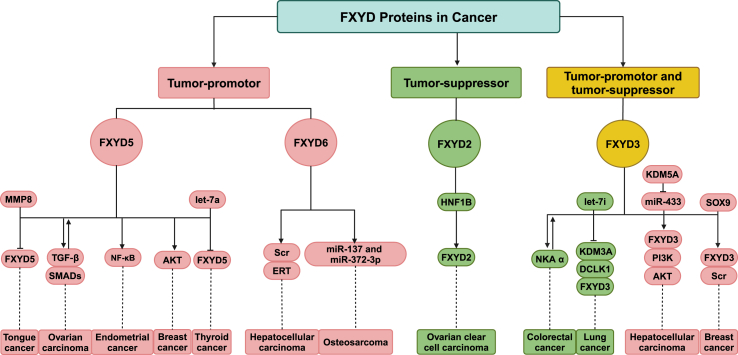
The tumor-promoting and tumor-suppressive functions of FXYD family proteins in various cancer types. FXYD5 and FXYD6 primarily exhibit oncogenic roles by influencing pathways such as TGF-β/SMAD, PI3K/AKT, and NF-κB. Conversely, FXYD2 acts as a tumor suppressor, particularly in ovarian clear cell carcinoma, where it is transcriptionally regulated by HNF1B. FXYD3 demonstrates tumor-promoting and tumor-suppressive roles: it promotes tumorigenesis in cancers like pancreatic cancer and bladder cancer, but exerts suppressive effects in certain conditions. The red denotes tumor-promoting activity, the green indicates tumor-suppressive function, and the yellow highlights proteins with both roles.

In ovarian carcinoma, FXYD5 overexpression correlates with poor prognosis and carboplatin resistance.^20^ It induces a mesenchymal phenotype, modulates chemokine production, and activates a TGF-β/SMAD feedback loop that promotes epithelial-to-mesenchymal transition and cancer progression. Reducing FXYD5 levels enhances cisplatin sensitivity while decreasing migration, invasion, and epithelial-to-mesenchymal transition.^71^ In tongue cancer, FXYD5 worsens prognosis by diminishing cell adhesion, contrasting with the adhesive effects of matrix metallopeptidase 8 (MMP8).^72^ In endometrial cancer, its expression correlates with TGF-β1 signaling and tumor aggressiveness. Study suggests that FXYD5 contributes to breast cancer progression by activating AKT signaling, promoting metastasis, epithelial–mesenchymal transition, cell motility, and drug resistance. Its overexpression is linked to poor prognosis and immune evasion.^73^ In breast cancer, it enhances cell motility and invasion.^73^^,^^74^ In a 4T1 breast cancer model, FXYD5 knockdown limited lung metastasis without affecting primary tumor growth, underscoring its role in metastasis.^5^ In thyroid cancer, an inverse correlation with let-7a levels suggests that FXYD5 supports cell adhesion and morphology, thereby reducing migration in thyroid follicular adenomas and carcinomas^75^ (Fig. 4).

FXYD6 is overexpressed in several cancers, including cholangiocarcinoma, HCC, and osteosarcoma. In cholangiocarcinoma, its expression correlates with better differentiation and may serve as a diagnostic biomarker.^62^ In HCC, FXYD6 up-regulation promotes cell migration and proliferation through enhanced Src/extracellular signal-regulated kinase (ERK) signaling, making it a potential therapeutic target; similar effects are observed in hepatitis B virus-related hepatocellular carcinoma.^63^^,^^64^ In osteosarcoma, FXYD6 facilitates tumor growth and migration by up-regulating miR-137 and miR-372-3p.^76^ It also plays a critical role in the immune dynamics of pediatric glioblastoma by influencing phenotypic transitions and tumor evolution.^77^ Moreover, WGCNA analysis has identified FXYD6 as a hub gene in pancreatic cancer, further demonstrating its clinical relevance^78^ (Fig. 4).

### Tumor-suppressive effects

FXYD2 primarily functions as a tumor suppressor across various cancers. It is highly expressed in several tumor types, including renal cancer and ovarian clear cell carcinoma (OCCC).^65^^,^^79^, ^80^, ^81^, ^82^, ^83^ In chromophobe renal cell carcinoma (RCC), FXYD2 overexpression serves as a diagnostic marker, distinguishing renal cell carcinoma from other renal tumors.^79^ In OCCC, high FXYD2 levels correlate with a favorable prognosis and are transcriptionally regulated by hepatocyte nuclear factor 1 homeobox B (HNF1B)^65^ (Fig. 4). Additionally, silencing FXYD2 triggers autophagic cell death and inhibits tumor growth, while elevated FXYD2 mRNA levels in gliomas enhance temozolomide (TMZ) sensitivity and are associated with improved patient survival.^21^

### Both tumor-promoting and tumor-suppressive effects

FXYD3 functions as either an oncogene or a tumor suppressor, depending on the cancer type. It primarily exhibits oncogenic properties in cancers such as breast cancer,^19^ colorectal cancer,^84^ pancreatic ductal adenocarcinoma,^4^ gastric cancer,^85^ liver cancer,^86^ bladder cancer,^18^ esophageal squamous cell carcinoma,^87^ gliomas,^88^ and endometrial cancer.^89^ However, in colon cancer, FXYD3 appears to act as a tumor suppressor, underscoring its context-dependent roles.^66^

FXYD3 overexpression in pancreatic cancer may drive tumor proliferation, highlighting its potential role in disease progression and therapeutic targeting. FXYD3 is identified as a key oncogene in the early stages of pancreatic cancer, with its expression correlating with enhanced migratory ability, highlighting its potential as a clinical biomarker for early detection and metastasis prediction.^4^^,^^67^

In colorectal cancer (CRC), FXYD3 interacts with the NKA α subunit, although a Gly41→Arg mutation disrupts this interaction.^90^ Its expression correlates with tumor stage, prognosis, and markers such as Ras, legumain, and proliferating cell nuclear antigen (PCNA), implicating it in tumor progression and metastasis.^91^^,^^92^ Differential responses to preoperative radiotherapy based on FXYD3 levels further highlight the need to understand its molecular mechanisms.^93^ Additionally, FXYD3 serves as a biomarker for bladder cancer staging and micrometastasis detection.^94^, ^95^, ^96^

In breast cancer, FXYD3 is highly expressed in both human and mouse tumors and is associated with estrogen receptor α (ERα), which regulates its expression. Estrogen and tamoxifen treatments elevate FXYD3 levels in ERα-positive cells, emphasizing the receptor's role. Inhibiting FXYD3 enhances sensitivity to doxorubicin and radiation, making it a potential target for chemosensitization or radiosensitization. Moreover, SRY-box transcription factor 9 (SOX9) promotes FXYD3 expression, which is crucial for SOX9 nuclear localization and the growth of estrogen receptor β (ERβ)-positive breast cancer stem cells^97^^,^^98^ (Fig. 4).

In HCC, FXYD3 expression is higher than in adjacent normal tissues. The lysine demethylase 5A (KDM5A)–miR-433–FXYD3–phosphoinositide 3-kinase (PI3K)–AKT axis has been identified, wherein KDM5A suppresses miR-433, leading to FXYD3 up-regulation and subsequent activation of the PI3K–AKT pathway to promote tumorigenesis. Targeting KDM5A's regulation of FXYD3 may offer new therapeutic opportunities^86^ (Fig. 4).

In lung cancer, FXYD3 expression is generally lower than in non-cancerous lung tissue. A somatic point mutation (g55c, D19H) has been identified, and forced expression of FXYD3b restores normal cortical actin distribution in deficient cells. Low FXYD3 levels correlate with high expression of lysine demethylase 3A (KDM3A) and doublecortin-like kinase 1 (DCLK1), suggesting a role in lung cancer progression.^9^^,^^99^

In urothelial carcinoma (UC), FXYD3 is exclusively expressed, aiding in its differentiation from renal cell carcinoma. In clear-cell renal cell carcinoma, elevated FXYD3 is associated with poor prognosis, driven by hypoxia and pro-tumor immune infiltrates. Additionally, LINC01503-mediated up-regulation of FXYD3 in cervical cancer promotes tumor progression by sponging miR-342-3p^85^^,^^87^^,^^89^^,^^100^^,^^101^ (Fig. 4).

### FXYD proteins in neurological disorders

Altered expression or dysfunction of FXYD proteins in the brain has been linked to various neurological diseases, including Rett syndrome (RTT), neuropathic pain, Parkinson's disease, and psychiatric disorders.

In RTT, a neurodevelopmental disorder caused by mutations in the Methyl-CpG binding protein 2 (MECP2) gene, reduced phosphorylation of FXYD1 may improve disease outcomes.^102^ FXYD1 is a direct target of MECP2, and studies have shown that dysregulating FXYD1 expression can rescue deficits in neuronal arborization and potassium homeostasis in MeCP2-deficient male mice.^102^^,^^103^ Furthermore, treatment with insulin-like growth factor-1 (IGF-1) has been found to down-regulate FXYD1 phosphorylation and improve behavioral deficits in a mouse model of RTT.^104^ IGF-1 treatment *in vitro* and in RTT mouse models significantly reduces FXYD1 mRNA and phosphorylated FXYD1 protein (p-FXYD1), correlating with improvements in behavior, motor coordination, and cognitive function.^104^

In inflammatory and neuropathic pain, FXYD2 has been implicated. Research suggests that FXYD2 interacts with the α1 subunit of NKA, negatively regulating its activity, depolarizing nociceptive neurons, and contributing to mechanical allodynia during peripheral inflammation.^105^ Loss of FXYD2, either constitutively in FXYD2^−/−^ mice or acutely in a neuropathic pain model, alleviates mechanical hypersensitivity induced by peripheral nerve lesions^106^ (Table 2). Additionally, recent studies have shown that SIX homeobox 2 (SIX2) functions as an anti-inflammatory factor in microglia, reducing neuroinflammation in Parkinson's disease by up-regulating FXYD2 and protecting dopaminergic neurons in the substantia nigra.^14^ These findings suggest that elevated FXYD2 activity may contribute to persistent hypersensitivity in both inflammatory and neuropathic pain, positioning FXYD2 as a potential regulator of neuropathic conditions affecting somatosensory nerves^107^ (Table 2).

**Table 2:** FXYD proteins and related diseases.

Organ	Disease	Regulatory Factor(s)	Pathway/Mechanism	Role in Disease	Ref
Brain	RTT	MECP2, IGF-1	Downregulating p-FXYD1 through IGF-1	Improves behavioral deficits, cognitive function, and neuronal plasticity	^102^ ^104^
Brain	Inflammatory & neuropathic pain	FXYD2, α1-NKA	Negative regulation of NKA, nociceptive neuron depolarization	Maintains mechanical allodynia	^105^ ^106^
Brain	Parkinson's disease	SIX2, FXYD2	Upregulating FXYD2 through SIX2	Protects dopaminergic neurons and reduces neuroinflammation	^14^ ^107^
Brain	Schizophrenia	FXYD6 (Chr 11q22-24)	Involving in neuron development and excitability	Potential susceptibility gene	108, 109, 110, 111, 112
Brain	Bipolar disorder	FXYD7	Enhancing the K^+^ affinity of neuronal NKA	Possibly involves in neuronal function regulation, associates with disease pathology	^113^
Heart	Ventricular arrhythmias	miR-151-5p, FXYD1	Downregulates miR-151-5p due to estrogen deficiency	Increases susceptibility to arrhythmias during myocardial infarction	^13^
Heart	Heart failure	FXYD1 (PLM), NKA, Na^+^/Ca^2+^ exchanger	Altered PLM expression and phosphorylation	Modulates contractility and reduces arrhythmic risk	^27^
Vascular system	Hypertension, vascular remodeling	FXYD5, NKA	Regulating NKA in renal tubular cells and affecting vascular smooth muscle migration	Contributes to blood pressure regulation and vascular remodeling	114, 115, 116
Vascular system	Atherosclerosis	FXYD6, EWSR1	Promoting FXYD6 expression by interacting with EWSR1, thereby enhancing NKA activity, increasing intracellular cholesterol accumulation	Aggravates atherosclerotic lesion development	^117^
Kidney	Autosomal dominant renal hypomagnesemia	FXYD2 (G41R mutation)	Affecting magnesium reabsorption in the distal convoluted tubule through mutant FXYD2	Reduces FXYD2 expression leads to hypomagnesemia and hypermagnesuria	^118^
Kidney	ARF	FXYD4	Suppressing ROMK and NKA activity	Reduces renal potassium excretion, leading to hyperkalemia	^8^;^119^
Kidney	ATN	FXYD4	Impairing local NKA activity and K^+^ handling	Impairs renal potassium excretion, exacerbating hyperkalemia	^120^
Kidney	Glomerulonephritis	FXYD5	Modulating NKA activity and promoting cell migration	Likely contribute to podocyte dysfunction and glomerular barrier disruption	^121^
Colon	HSCR	FXYD1, Scn1b	Impacting ion channel function and electrical coupling	Contributes to impaired colonic motility and enteric nervous system dysfunction	^122^
Intestine	Bacterial infection	FXYD3, ExoS	Facilitating bacterial penetration and weakening tight junctions because ExoS binds to FXYD3	Disrupts intestinal epithelial barrier function and increases susceptibility to infection	^123^
Colon	Chronic diarrhea, IBD	FXYD4, corticosteroids	Dysregulation of Na^+^/K^+^ ion transport (corticosteroid-regulated)	Contributes to electrolyte imbalance and fluid retention issues	^124^;^125^
Skeletal muscle	Exercise adaptation	FXYD1, PGC-1α, AMPK	Increasing FXYD1 and PGC-1α expression under blood flow restriction; AMPK activation	Regulates NKA activity and K^+^ homeostasis during intense exercise	126, 127, 128
Lung	ARDS	FXYD1; TGF-β	Limiting NKA activity	Impairing alveolar fluid clearance and contributing to pulmonary edema	^129^
Pancreas	Diabetes	FXYD2	Increases β-cell mass and restoring Akt phosphorylation due to the loss of FXYD2	Potential therapeutic target for diabetes interventions	^130^
Pancreas	Glucose metabolism	FXYD3	Epigenetic regulation of FXYD3 promoter by CpG methylation	Suppresses insulin secretion, affecting glucose homeostasis	^131^
Skin	Psoriasis	FXYD3, IL-17A	Promoting IL-17R-ACT1 complex formation, activating NF-κB and MAPK pathways	Enhances pro-inflammatory cytokine expression	^132^
Lung	Inflammatory lung injury	FXYD5, NF-κB	Activating NF-κB, increasing cytokine production and monocyte recruitment	Promotes lung inflammation	^133^
Pancreas	Acute pancreatitis	FXYD5, JAK2/STAT3	JAK2/STAT3 pathway inhibition	Protects against pancreatitis-induced inflammation	^134^
Cartilage	Chondrocyte inflammation	FXYD5, NF-κB	NF-κB-mediated inflammation and extracellular matrix degradation	Exacerbates cartilage damage; reversible with NF-κB inhibitor	^57^

FXYD6, primarily expressed in the brain, plays a role in neuronal development and excitability. Its genetic locus on chromosome 11q22-24 has been linked to childhood-onset schizophrenia susceptibility, although its association with the disorder remains a subject of debate.^108^, ^109^, ^110^, ^111^, ^112^

In the context of bipolar disorder, chronic treatment with carbamazepine has been shown to differentially regulate NKA subunits and the auxiliary protein FXYD7 in neurons and astrocytes. Specifically, FXYD7 expression is down-regulated in neurons, which may enhance the K^+^ affinity of neuronal NKA and facilitate intracellular K^+^ uptake during periods of elevated extracellular K^+ 113^. This neuron-specific modulation, alongside carbamazepine-induced up-regulation of α2 and β1 subunits, likely contributes to improved potassium homeostasis and neuronal excitability control, suggesting that targeting FXYD7 could be of therapeutic relevance in managing ion imbalance-related aspects of bipolar disorder^113^ (Table 2).

Future research should further investigate the specific mechanisms by which the FXYD proteins contribute to psychiatric and neurological diseases and evaluate their potential as diagnostic biomarkers and therapeutic targets.

### FXYD proteins in cardiovascular diseases

Dysregulation of FXYD proteins has been implicated in various cardiovascular diseases, including myocardial infarction, heart failure, arrhythmias, hypertension, and atherosclerosis. For example, estrogen deficiency increases vulnerability to ventricular arrhythmias during myocardial infarction by down-regulating miR-151-5p, which in turn up-regulates FXYD1 and disrupts ion-channel function.^13^ In cardiomyocytes, FXYD1 (also known as phospholemman, PLM) fine-tunes ion homeostasis by modulating NKA, the Na^+^/Ca^2+^ exchanger, and L-type Ca^2+^ channels^13^ (Table 2). Phosphorylation of PLM at serine 68 enhances NKA activity while inhibiting the Na^+^/Ca^2+^ exchanger, thereby balancing intracellular Na^+^ and Ca^2+^ levels.^13^ In heart failure, alterations in PLM expression and phosphorylation may disrupt excitation-contraction coupling. Notably, PLM helps reduce arrhythmic risk and preserve cardiac contractility under stress, making it a promising target for heart failure drug development. The coordinated regulation of FXYD1 during cardiac stress is crucial for limiting arrhythmogenesis and sustaining myocardial contractility.^27^

Beyond its role in cardiac function, FXYD5 has emerged as a key regulator of blood pressure and vascular remodeling by modulating NKA activity in renal and vascular tissues.^114^, ^115^, ^116^ In spontaneously hypertensive rats, FXYD5 mRNA expression is significantly reduced in both mesenteric arteries and kidneys compared with normotensive controls, correlating with peak blood pressure levels. Mechanistically, FXYD5 knockdown in vascular smooth muscle cells inhibits cell migration and suppresses NKA activity, while FXYD5 overexpression in renal tubular epithelial cells enhances NKA function and promotes cellular proliferation, indicating tissue-specific regulatory roles.^114^, ^115^, ^116^ These findings suggest that FXYD5 contributes to hypertension not only through impaired renal sodium handling but also by influencing vascular structural remodeling via vascular smooth muscle cell motility. Furthermore, considering the role of mineralocorticoid receptor (MR) signaling in promoting inflammation and oxidative stress during vascular remodeling, it is plausible that FXYD5-NKA interactions may intersect with MR pathways, although this remains to be fully elucidated.^114^, ^115^, ^116^ Given its dual influence on renal epithelial and vascular smooth muscle physiology, FXYD5 represents a promising therapeutic target for modulating NKA activity in the context of hypertension, atherosclerosis, and MR-mediated vascular dysfunction (Table 2).

Furthermore, FXYD6 has been implicated in atherosclerosis through a regulatory axis involving the lncRNA RP11-728F11.4 and Ewing's sarcoma RNA binding protein-1 (EWSR1). RP11-728F11.4 promotes FXYD6 expression by interacting with EWSR1, leading to enhanced NKA activity, increased intracellular cholesterol accumulation, and elevated proinflammatory cytokine production in macrophages.^117^ *In vivo* studies confirm that this pathway aggravates atherosclerotic lesion development, suggesting that disrupting this axis could offer novel strategies to combat vascular inflammation and atherosclerosis^117^ (Table 2).

### FXYD proteins in kidney diseases

FXYD proteins play a vital role in regulating ion homeostasis, especially in the kidneys, where precise control of sodium, potassium, and chloride ions is crucial for maintaining fluid balance, blood pressure, and overall renal function. Alterations in the expression or function of FXYD proteins have been implicated in various kidney diseases, including autosomal dominant renal hypomagnesemia, acute renal failure (ARF), acute tubular necrosis (ATN), and glomerulonephritis.

Autosomal dominant renal hypomagnesemia, often associated with hypocalciuria, has been linked to a G41R mutation in the FXYD2 gene (121G > A) within the TM domain.^118^ It has been reported that FXYD2 expression in proximal tubular cells, isolated from both a patient and a healthy subject, is lower in the patient's cells, with both FXYD2a and FXYD2b mRNA carrying the mutation.^135^ Further studies have confirmed that FXYD2 (G41R) mutations are associated with hypomagnesemia and hypermagnesuria.^136^ Notably, these mutations specifically affect the distal convoluted tubule^137^, ^138^, ^139^ (Table 2).

In ischemia-reperfusion-induced acute renal failure, FXYD4 expression is significantly down-regulated in the renal cortex and medulla, coinciding with suppressed renal outer medullary K^+^ and NKA activity, which impairs renal potassium secretion and contributes to hyperkalemia.^119^ Conversely, FXYD4 is markedly up-regulated in the distal colon, potentially mediated by aldosterone, serving as a compensatory mechanism for colonic K^+^ excretion.^119^ These findings support a functional role for FXYD4 in modulating epithelial potassium transport under ARF conditions and suggest therapeutic potential in targeting extrarenal FXYD4 expression to mitigate hyperkalemia.^8^^,^^119^ In glycerol-induced acute tubular necrosis, FXYD4 (CHIF) mRNA is profoundly decreased in the renal medulla and papilla in proportion to the severity of tubular injury and hyperkalemia, likely impairing local NKA activity and K^+^ handling. Meanwhile, CHIF expression in the colon increases significantly in a severity- and aldosterone-dependent manner, although this up-regulation fails to fully correct systemic hyperkalemia.^120^ These data reinforce the central role of renal FXYD4 in potassium homeostasis and suggest that therapeutic strategies aimed at preserving or enhancing its expression could be beneficial in acute tubular necrosis-associated electrolyte disturbances^120^ (Table 2).

Additionally, FXYD5 has been identified in podocytes from glomerulonephritis patients through single-cell RNA sequencing, suggesting a potential role in disease-specific podocyte injury. Given its known involvement in modulating NKA activity and promoting cell migration in other tissues, FXYD5 may contribute to podocyte dysfunction and glomerular barrier disruption^121^ (Table 2). However, its precise function in renal pathology remains to be elucidated, warranting further investigation into its potential as a biomarker or therapeutic target in glomerulonephritis.

### FXYD proteins in gastrointestinal diseases

FXYD proteins play a critical role in maintaining cellular ion balance, and their dysregulation has been associated with various gastrointestinal disorders, particularly in regulating intestinal ion transport, gastric acid secretion, and overall gastrointestinal function. It has been reported that sodium channel type 1β (Scn1b), the predominant sodium channel gene in murine colonic cells, is highly expressed alongside FXYD1.^140^ Moreover, studies have demonstrated significantly down-regulated FXYD1 expression in both aganglionic and ganglionic segments of Hirschsprung colon tissue compared with controls. Given that FXYD1 is expressed in both interstitial cells of Cajal (ICC) and PDGFRα^+^ cells (key components of the SIP syncytium involved in coordinating smooth muscle contraction), its down-regulation may contribute to persistent motility dysfunction even in the resected ganglionic bowel.^141^ These findings suggest that altered FXYD1 expression may impact ion channel function and electrical coupling within the enteric neuromuscular apparatus, influencing functional outcomes in patients with Hirschsprung disease despite anatomically normal ganglion cell presence.^122^

FXYD3 has been implicated in intestinal epithelial barrier defense. The bacterial effector protein, encoding the type III effector protein, ExoS, produced by *Pseudomonas aeruginosa*, binds to FXYD3, facilitating bacterial penetration and weakening tight junctions, thereby compromising epithelial integrity.^123^

Abnormal FXYD4 expression in the colon,^8^ often influenced by corticosteroid levels, can disrupt sodium and potassium ion transport, contributing to electrolyte imbalances and water retention issues. This dysfunction has been associated with conditions such as chronic diarrhea and inflammatory bowel disease (IBD), where impaired ion transport and fluid balance exacerbate disease symptoms. Insufficient FXYD4 expression may reduce the colon's ability to absorb electrolytes effectively, further aggravating these gastrointestinal disorders.^124^^,^^125^

### FXYD proteins in other diseases

FXYD1 is implicated in exercise-induced muscle adaptations. Blood flow restriction exercise increases FXYD1 and peroxisome proliferator-activated receptor-γ coactivator-1α (PGC-1α) mRNA, potentially via oxidative stress and AMP-activated protein kinase (AMPK) signaling.^141^ FXYD1 protects against oxidative damage by reversing NKA β1 subunit S-glutathionylation and potentially preventing endothelial NO synthase (eNOS) oxidation.^39^ It also influences muscle NKA activity and potassium balance during intense exercise.^126^, ^127^, ^128^, ^142^ In acute respiratory distress syndrome (ARDS), FXYD1 is up-regulated in alveolar epithelial cells, potentially downstream of TGF-β signaling, a key pathogenic factor in acute respiratory distress syndrome. Functional studies suggest that FXYD1 overexpression reduces NKA-mediated ion transport, thereby impairing alveolar fluid clearance and contributing to pulmonary edema.^129^

FXYD2 may play an important role in diabetes by regulating pancreatic β-cell growth. In Fxyd2 knockout mice, β-cell hyperplasia and increased insulin levels are observed, contributing to improved glucose tolerance without peripheral insulin resistance. Mechanistically, FXYD2 suppresses Akt phosphorylation in β-cells, a key signaling event for cell proliferation; this inhibition is reversed upon FXYD2 degradation in INS 832/13 cells. These findings suggest that FXYD2 negatively regulates β-cell mass through the Akt pathway, and its modulation may represent a potential therapeutic strategy to enhance endogenous insulin production in diabetes.^130^

FXYD3 epigenetically regulates insulin secretion in pancreatic β-cells. Its promoter methylation suppresses transcription, while hypomethylation and overexpression, observed in diabetic islets, impair glucose-stimulated insulin secretion, linking FXYD3 to β-cell dysfunction in type 2 diabetes.^131^ FXYD3 contributes to inflammation, enhancing interleukin-17A (IL-17A) signaling in keratinocytes, activating nuclear factor kappa B subunit (NF-κB) and mitogen-activated protein kinase (MAPK) pathways, and potentially worsening psoriasis.^132^

FXYD5 is involved in inflammation in multiple contexts. Its overexpression in epithelial cells increases C–C motif ligand 2 (CCL2) levels.^143^ In lung epithelial cells, FXYD5 activates NF-κB, increasing cytokine production and monocyte recruitment in lung injury.^133^ Reducing FXYD5 in acute pancreatitis decreases inflammation via the Janus kinase 2 (JAK2)/signal transducer and activator of transcription 3 (STAT3) pathway.^134^ FXYD5 also promotes inflammation and extracellular matrix breakdown in chondrocytes, reversible with NF-κB inhibitors.^57^

## Conclusions and future perspectives

FXYD proteins are crucial for cellular ion homeostasis and NKA modulation, impacting diverse physiological processes.^14^, ^15^, ^16^, ^17^ Their dysregulation is linked to various diseases, making them promising therapeutic targets.^27^^,^^61^^,^^70^^,^^77^

Modulating FXYD activity shows potential for restoring cellular function. Targeting FXYD1 may improve cardiac function in cardiovascular diseases.^27^ Modulating FXYD2 and FXYD4 could aid electrolyte management in renal diseases.^139^^,^^119^ FXYD6 and FXYD7 are potential targets for neurological disorders,^108^^,^^113^ while FXYD3 and FXYD5 are implicated in various types of cancer.^71^^,^^72^^,^^88^^,^^144^

Future research should focus on the precise molecular mechanisms of FXYD involvement in diseases. Investigating FXYD-NKA interactions and post-translational modifications is crucial. Developing gene therapies, small-molecule inhibitors, and peptide-based treatments is key. Clinical trials are essential to validate the safety and efficacy of FXYD-targeted therapies.

In summary, FXYD proteins offer therapeutic potential, but further research is needed. Future work should refine our understanding of FXYD functions, develop selective agents, and address clinical challenges. Continued investment in this area may lead to new treatments and improved patient outcomes.

## CRediT authorship contribution statement

**Xi Li:** Writing – review & editing. **Min Long:** Writing – review & editing. **Shangwei Zhong:** Writing – review & editing. **Jun-Li Luo:** Writing – review & editing.

## Data availability

All data generated or analyzed during this study are included in this published article.

## Funding

This work was supported by the grants from the 10.13039/501100001809National Natural Science Foundation of China (No. 81974469, 82472835), the National Science Foundation of China for Youths (No. 82203166), General Project of Hunan Provincial Health Commission Research Program (China) (No. B202302057895), the Science and Technology Innovation Program of Hunan Province, China (No. 2022RC4044), the Hunan Province Graduate Research and Innovation Project (China) (No. CX20230951, CX20230952), and the Foundation of Hunan Provincial Key Laboratory (China) (No. 2023TP1014).

## Conflict of interests

The authors declared no conflict of interests.
